# Recombinant Thrombomodulin Exerts Anti-autophagic Action in Endothelial Cells and Provides Anti-atherosclerosis Effect in Apolipoprotein E Deficient Mice

**DOI:** 10.1038/s41598-017-03443-z

**Published:** 2017-06-12

**Authors:** Po-Sheng Chen, Kuan-Chieh Wang, Ting-Hsing Chao, Hsing-Chun Chung, Shi-Ya Tseng, Chawn-Yau Luo, Guey-Yueh Shi, Hua-Lin Wu, Yi-Heng Li

**Affiliations:** 1 Department of Internal Medicine, National Cheng Kung University Hospital and College of Medicine, Tainan, Taiwan; 2 Department of Biochemistry and Molecular Biology, National Cheng Kung University Hospital and College of Medicine, Tainan, Taiwan; 3 Department of Surgery, National Cheng Kung University Hospital and College of Medicine, Tainan, Taiwan; 4 Institute of Clinical Medicine, National Cheng Kung University Hospital and College of Medicine, Tainan, Taiwan; 5 Department of Tourism Management, College of Recreation and Health Management, Chia Nan University of Pharmacy and Science, Tainan, Taiwan

## Abstract

Stress-induced alteration in endothelial cells (ECs) integrity precedes the development of atherosclerosis. Previous studies showed that the soluble recombinant thrombomodulin (rTM) not only increases ECs proliferation but also exerts anti-apoptotic activity in ECs. However, the functional significance of soluble rTM on autophagy-related apoptosis in ECs is still undetermined. Implicating a cytoprotective role for rTM in persistent serum starvation (SS)-induced autophagy in cultured ECs, we found that treatment of rTM decreased the expression of SS-induced autophagy-related proteins, ATG5 and LC3, and the formation of autophagosomes through activation of AKT/mTOR pathway. In addition, treatment of rTM decreased SS-induced EC apoptosis, but this effect of rTM could not be recapitulated by co-treatment with a potent autophagy inducer, rapamycin and in ECs with ATG5 knockdown. In human atherosclerosis specimens, expression of autophagy markers, ATG13 and LC3, were more abundant in aortic intimal ECs with severe atherosclerosis than those without atherosclerosis. Moreover, compared to saline treatment group, administration of rTM reduced LC3 and ATG13 expression, intimal EC apoptosis, and atherosclerotic lesion severity in the aorta of apolipoprotein E deficient mice. In conclusion, treatment with rTM suppressed stress-induced autophagy overactivation in ECs, provided ECs protective effects, and decreased atherosclerosis in apolipoprotein E deficient mice.

## Introduction

Endothelial cells (ECs) form the inner layer of vascular wall and involve in critical physiological processes, such as vascular tone modulation, blood coagulation, and inflammation. Exposure of ECs to stress results in loss of their integrity and function, and precedes the development of atherosclerosis^1^. Autophagy is an evolutionarily conserved mechanism for controlled degradation of proteins, macromolecules, and organelles during stress such as nutrient starvation and hypoxia^2, 3^. After activation, autophagy starts with the formation of autophagosomes with sequestrated molecules and then fuses with lysosomes to degrade the contents. It is generally recognized as a cell pro-survival mechanism responsive to stress by recycling of amino acids, free fatty acids or nucleotides derived from the degradation process. However, if the cellular stress is overwhelming or persistent, excessive induction of autophagy becomes an alternative mechanism for programmed cell death^4, 5^. One of the major signaling pathways to regulate autophagy is through phosphatidylinositol 3-kinase (PI3K)–protein kinase B/AKT/mammalian target of rapamycin (mTOR) complex 1 pathway. The mTOR complex 1 negatively regulates a complex consisting autophagy related proteins (ATGs) that initiate autophagosome formation^6^. Recent study found that transient exposure of ECs to hypoxia may induce autophagy to promote ECs survival and growth, but if the hypoxia stress was prolonged, autophagy became mTOR dependent and persistently activated autophagy induced ECs apoptosis^7^. Vascular ECs are exposed to circulating inflammatory mediators that may trigger autophagy. During atherosclerotic process, deprivation of oxygen and nutrient from insufficient vascularization of growing neointima or atherosclerotic plaque causes persistent autophagy activation in the arterial wall^8^. Hence, targeting ECs death induced by overactivated autophagy and restoring endothelial dysfunction might be considered to be a potential therapeutic strategy for preventing atherosclerosis development.

Thrombomodulin (TM) is a cell membrane-bound glycoprotein mainly expressed on ECs. It consists of 5 domains including a highly charged N-terminal lectin-like domain (D1), a domain with six epidermal growth factor (EGF)-like structures (D2), a serine and threonine-rich domain (D3), a transmembrane domain (D4) and a cytoplasmic tail (D5). TM binds to thrombin via its EGF-like domain and activates protein C (APC) which degrades coagulation factors VIIIa and Va^9^. After binding thrombin, TM decreases the thrombin’s procoagulant effects. Thrombin activates protease-activated receptor 1 (PAR-1)/sphingosine-1 phosphate receptor 3 pathway in ECs and causes junction disruption, adhesion molecule expression and monocyte adhesion on ECs^10^. All the proinflammatory responses induced by thrombin are also reduced by TM. The membrane-bound TM is released from ECs during stress and becomes soluble form in systemic circulation. Soluble TM has the similar ability to inhibit thrombin and produce APC as its membrane-bound form. Originally, soluble TM is considered only as a marker of endothelial damage. Later studies showed that soluble recombinant TM (rTM) confers direct ECs protective effect by decreasing stress-induced ECs apoptosis and increasing ECs proliferation^11, 12^. Our previous studies demonstrated that the cytoprotective effects of rTM are independent of its thrombin inhibition or APC producing functions, and works by direct binding of rTM to fibroblast growth factor receptor 1 (FGFR1) on ECs with subsequent PI3K/AKT pathway activation^12–14^ which is also important in inhibition of autophagy. In the current study, we hypothesized that: (1) rTM is an autophagy inhibitor by activating PI3K/AKT/mTOR complex 1 signaling pathway; (2) rTM suppresses stress-induced autophagy overactivation in ECs to provide ECs protective effects; and (3) rTM treatment may suppress overactivated autophagy in ECs during the process of atherosclerosis formation and decrease atherosclerosis severity in animal atherosclerosis model. The following experiments were designed to prove our hypotheses.

## Results

### rTM suppresses stress-induced autophagy in cultured ECs

**Figure 1:**
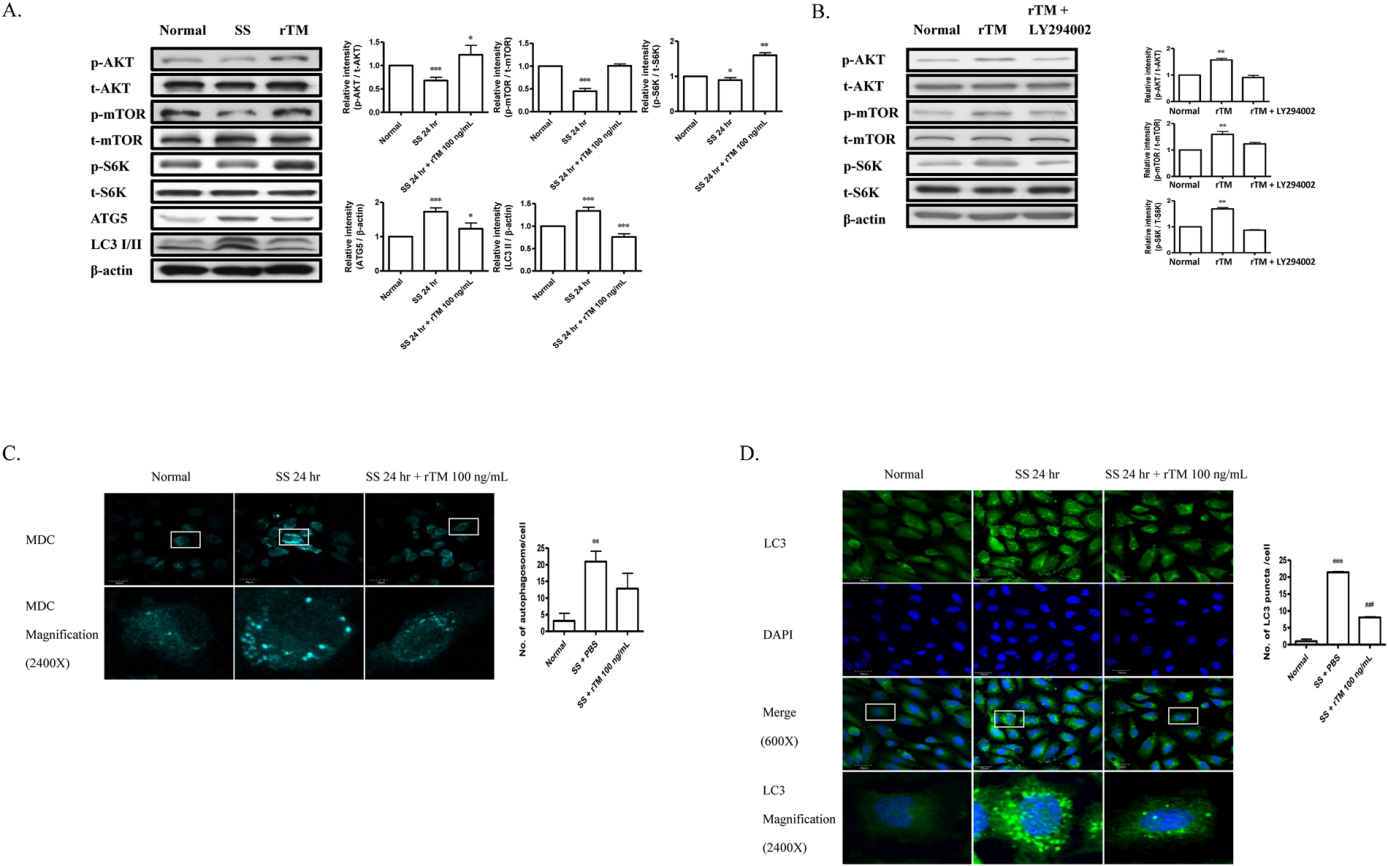
rTM suppresses serum starvation-induced autophagy in ECs. (**A**) Western blot analyses were performed in ECs without SS (Normal), with SS (SS) or SS + rTM 100 ng/mL (rTM). Bar graphs showed the ratios of protein expression levels at 24 hr in ECs of SS or rTM group to normal group. Data are shown as mean ± SEM (n = 3). *p < 0.05, **p < 0.01 and ***p < 0.001 vs. cells without SS (Normal). (**B**) Western blot analyses were performed in ECs treated with rTM 100 ng/mL or rTM + AKT inhibitor, LY294002 10 mM for 24 hr. Bar graphs showed the ratios of protein expression levels in cells with rTM or rTM + LY294002 to saline treatment (Normal). Data are shown as mean ± SEM (n = 3). **p < 0.01 vs. cells with saline treatment (Normal). (**C** and **D**) Immunofluorescence staining of autophagosomes with MDC (**C**) and LC3 (**D**) in cultured ECs with normal condition without SS (Normal), with SS + phosphate buffered saline (PBS) and with SS + rTM 100 ng/mL treatment. DAPI was used to stain the nuclei of ECs. Magnified view (2400X) of a representative cell was shown. Bar = 30 μm. Bar graphs showed the quantification of MDC-positive dot staining (**C**) or LC3 puncta per cell (**D**). Ten cells were counted and averaged for each labeling condition. Data are mean ± SEM. **p < 0.01, ***p < 0.001 compared with cells under normal condition without SS. ^###^p < 0.001 compared with cells under SS for 24 hr.

We stressed the human umbilical vein endothelial cells (HUVECs) with serum starvation (SS, 0.5% fetal bovine serum [FBS]) for 24 hr. SS decreased the phosphorylation of AKT, mTOR and the downstream effector protein, p70 ribosomal S6 kinase (S6K). Autophagy-related proteins, including ATG5 and microtubule-associated protein 1 light chain 3 (LC3) expression were increased during starvation (Fig. 1A). Treatment with rTM 100 ng/mL restored AKT, mTOR and S6K phosphorylation and inhibited ATG5 and LC3 expression at 24 hr after starvation (Fig. 1A). AKT inhibitor, LY294002, significantly reduced rTM effect on mTOR and S6K phosphorylation indicating that rTM effect on mTOR works through AKT activation (Fig. 1B). Monodansylcadaverine (MDC) (Fig. 1C) and LC3 (Fig. 1D) staining showed that persistent SS for 24 hr significantly induced autophagosomes formation in ECs and rTM 100 ng/mL treatment could decrease the number of autophagosomes in ECs. These results demonstrated that rTM treatment suppressed stress-induced autophagy overactivation in ECs.

### Autophagy plays a role in rTM effect on ECs

**Figure 2:**
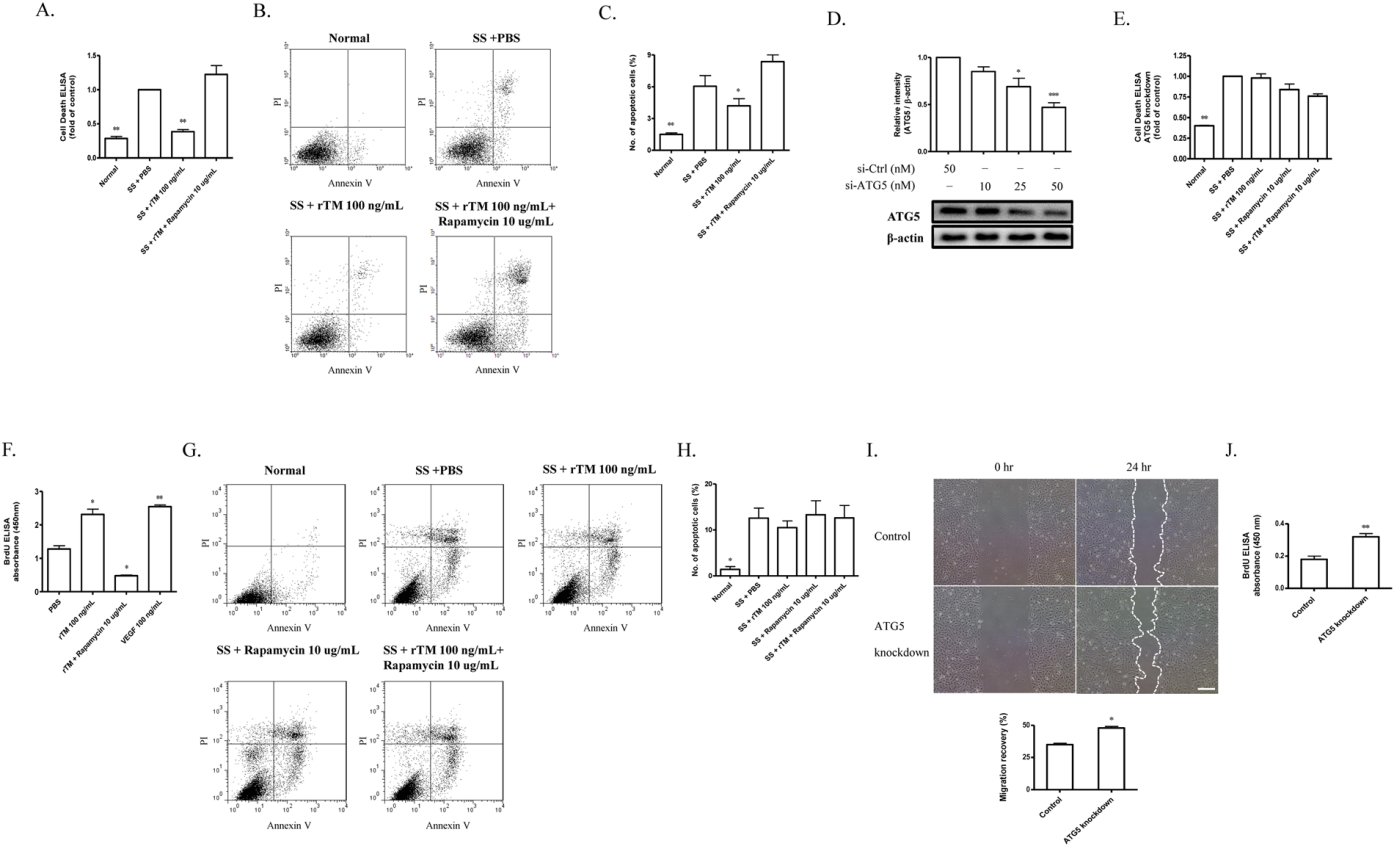
Autophagy plays a role in rTM effect on ECs. (**A** and **E**) Normal (**A**) and ATG5 knockdown (**E**) ECs received various indicated treatment. Apoptosis was determined with a cell death detection ELISA kit. The data were expressed as absorbance ratios of various indicated treatment to SS + PBS treatment. Data are shown as mean ± SEM (n = 3). **p < 0.01 vs. cells with SS + PBS treatment. (**B** and **C**; **G** and **H**) Apoptosis in normal (**B**) and ATG5 knockdown ECs (**G**) was also determined with a FITC annexin V apoptosis detection kit with flow cytometry. The representative pictures from each group were shown (**B** and **G**). The data were expressed as the percentage of apoptotic cell count (Annexin V-FITC positive and PI positive)/total cell count (**C** and **H**). All results were obtained from three independent experiments. Data are shown as mean ± SEM (n = 3). *p < 0.05 and **p < 0.01 vs. cells with SS + PBS treatment. (**D**) ECs were transfected with control siRNA (si-Ctrl) and ATG5 siRNA (si-ATG5). The knockdown efficiency of ATG5 in ECs was measured by western blot. Data are shown as mean ± SEM (n = 3). *p < 0.05 and ***p < 0.001 vs. cells with si-Ctrl transfection. (**F**) Cell proliferation was measured in ECs received various indicated treatment. Data were expressed as BrdU absorbance and shown as mean ± SEM (n = 3). *p < 0.05, **p < 0.01 vs. cells with PBS treatment. VEGF was used as a positive control. (**I** and **J**) Migration and proliferation ability were compared between ECs with and without ATG knockdown (control transfection). Representative photomicrographs of the ECs migration during wound healing assay were shown (**I**; left panel). Cultured ECs were wounded with pipette tips. The migration ability was expressed by the percentage of distance at 24 hr/0 hr (**I**; right panel). Bar = 500 μm. (**J**) Proliferation ability was measured with BrdU assay. Data are shown as mean ± SEM (n = 3). *p < 0.05, **p < 0.01 vs. cells without ATG5 knockdown (control).

Cell death detection ELISA showed that SS for 24 hr induced ECs apoptosis. There was a significant reduction of apoptosis in rTM-treated ECs after SS. Rapamycin (a potent autophagy activator) treatment abolished the rTM protective effect on SS-induced ECs apoptosis (Fig. 2A). Flow cytometry study with annexin V and propidium iodide staining reconfirmed the findings that rTM treatment reduced SS-induced EC apoptosis and rapamycin abolished the rTM effect (Fig. 2B and C). ATG 5 knockdown ECs do not have significant autophagy after stress^15^. To investigate whether inhibition of autophagy could abolish rTM cytoprotective effect on ECs, ATG5 knockdown ECs were used. The efficacy of ATG5 knockdown was checked by western blot (Fig. 2D). In ATG 5 knockdown ECs, we found SS could induce cell apoptosis, but rTM or rapamycin treatment could not influence SS-induced cell apoptosis shown by cell death ELISA (Fig. 2E) and flow cytometry (Fig. 2G and H). Treatment with rTM induced EC proliferation. Autophagy overactivation caused by rapamycin also inhibited rTM-induced ECs proliferation (Fig. 2F). Wound healing and BrdU assays showed that ECs with ATG5 knockdown had higher migration and proliferation ability than ECs without ATG5 knockdown (Fig. 2I and J). These results indicated that autophagy played an important role in rTM protective effect on stress-induced EC apoptosis and EC behaviors through AKT/mTOR pathway.

### Autophagy is activated in intimal ECs in human atherosclerosis

**Figure 3:**
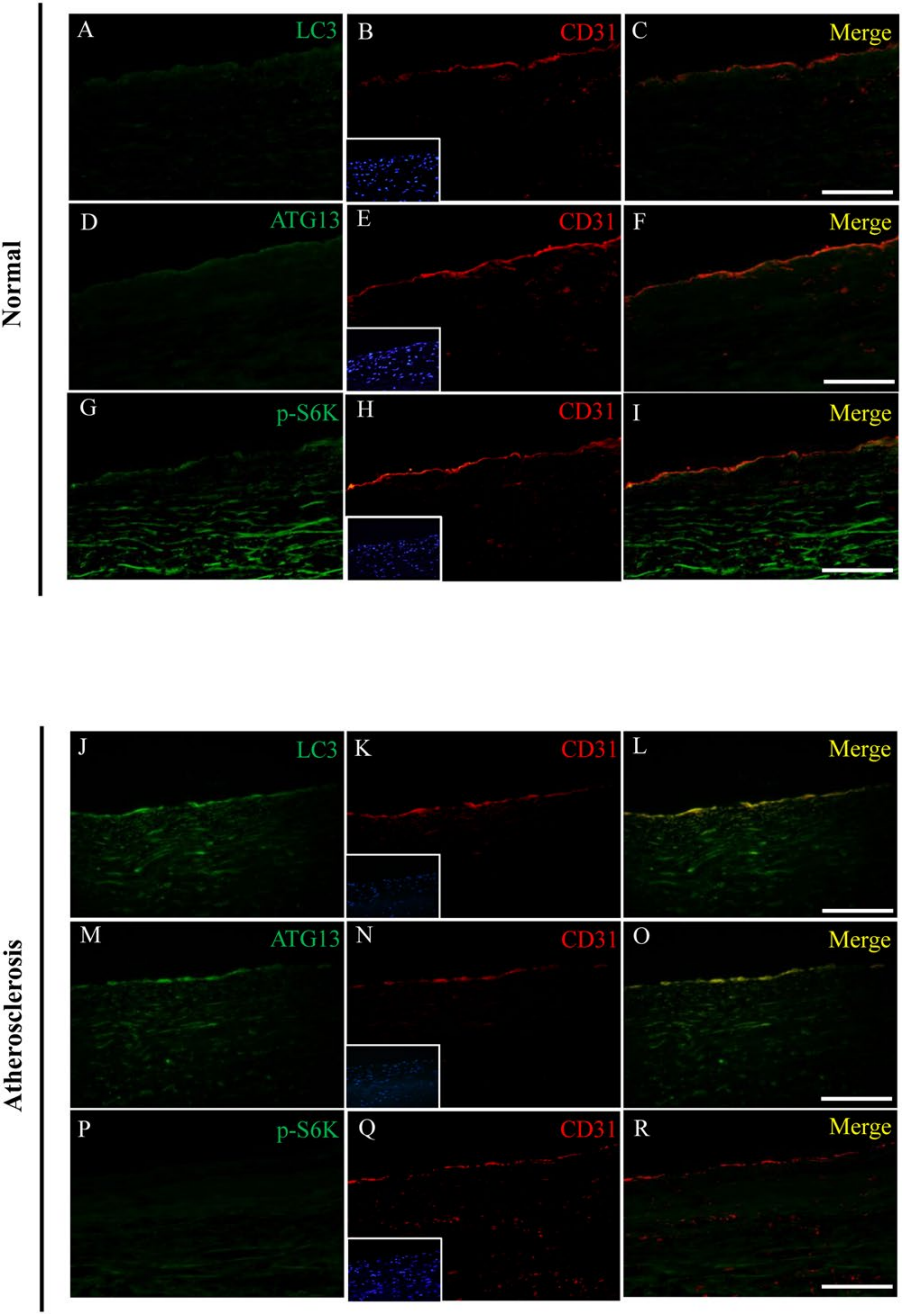
Autophagy is activated in intimal ECs in human atherosclerosis. Representative microscopic images of double immunofluorescent staining for LC3, ATG13 and p-S6K with CD31 (a marker for ECs) in the aortic specimens from a healthy cardiac donor (**A**–**I**) and a recipient (**J**–**R**) of heart transplantation for ischemic cardiomyopathy. Nuclei were counterstained with DAPI. Bar = 200 μm.

In human aortic specimens, we investigated the expression of autophagy-related proteins, LC3 and ATG13 in aortic ECs in patients with or without severe coronary atherosclerosis. The signals of LC3 and ATG13 were low in intimal CD31-positive ECs in patient without significant atherosclerosis (Fig. 3A–F); while the p-S6K signal was found (Fig. 3G–I). But in patient with severe coronary atherosclerosis, double immunofluorescent staining revealed that LC3 and ATG13 were highly expressed in aortic intimal ECs (Fig. 3J–O) and no p-S6K signal can be found (Fig. 3P–R). The findings indicated that autophagy was activated in intimal ECs in human atherosclerosis.

### rTM suppresses ECs autophagy activation in apolipoprotein (apo)E deficient mice

**Figure 4:**
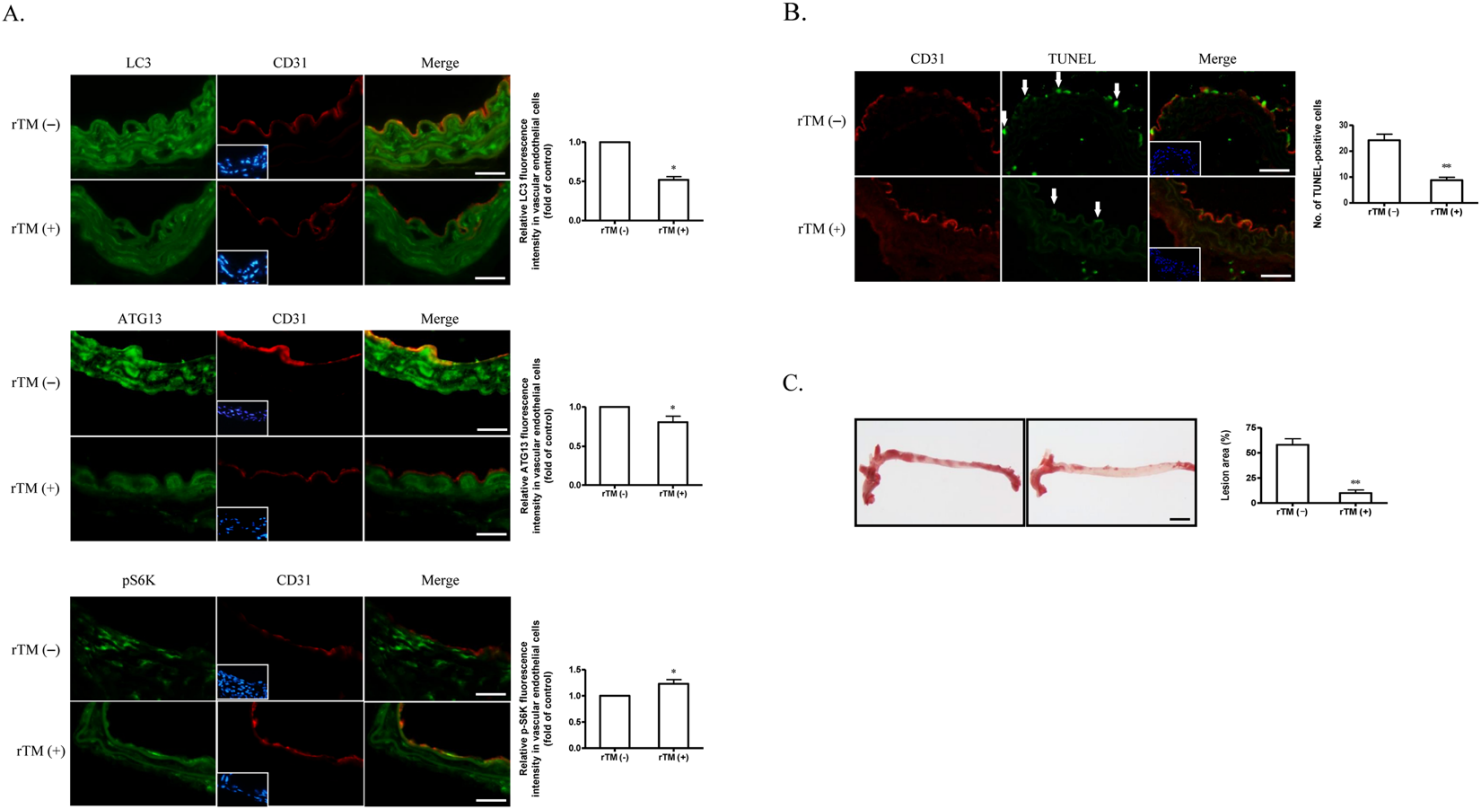
rTM suppresses ECs autophagy activation in apoE deficient mice. (**A**) Representative microscopic images of double immunofluorescent staining for LC3 (upper panel), ATG13 (middle panel), or p-S6K (lower panel) with CD31 (a marker for ECs) in the aortic specimens from apoE deficient mice. Nuclei were counterstained with DAPI. Bar = 50 μm. Bar graphs showed the ratios of double staining fluorescent intensity in the mice with rTM treatment to mice without rTM treatment. Data are mean ± SEM. Three aortic sections were stained and measured. *p < 0.05 compared with the mice without rTM treatment. (**B**) Representative microscopic images of double immunofluorescent TUNEL and CD31 staining in the aortic specimens from apoE deficient mice. Nuclei were counterstained with DAPI. Bar = 50 μm. Apoptotic cells (arrows) in aortic intimal layer were quantified by counting and averaging the number of TUNEL-positive cells in 5 aortic sections. Bar graph showed the apoptotic ECs in mice with and without rTM treatment. Data are mean ± SEM. **p < 0.01 vs. mice without rTM treatment. (**C**) Representative images of aortas with Oil-red O staining from apoE deficient mice with (right) and without (left) rTM treatment. Bar = 500 μm. The atherosclerotic severity was expressed as a percentage of atherosclerotic plaque area to the total aortic surface area. Bar graph showed the atherosclerotic severity in mice with and without rTM treatment. Data are mean ± SEM (n = 3). **p < 0.01 vs. mice without rTM treatment.

In order to determine whether treatment of rTM inhibits ECs autophagy overactivation *in vivo*, we used a well-established mouse model, apoE deficient mice fed with high fat diet, to recapitulate the development of human atherosclerosis^16^. In apoE deficient mice without rTM treatment, double immunofluorescent staining revealed that LC3 and ATG13 were highly expressed in aortic intimal ECs. Treatment with rTM reduced the signals of LC3 and ATG13 in aortic intimal ECs and increased the signal of mTOR downstream effector protein p-S6K (Fig. 4A). TUNEL assay was used to evaluate cell apoptosis. The number of TUNEL-positive cells in intimal layer was significantly reduced in rTM-treated mice (Fig. 4B). ApoE deficient mice with rTM treatment had less atherosclerotic plaque lesions (with vs. without rTM treatment, Oil Red stained aortic plaque area: 11 ± 4% vs. 57 ± 7%, p < 0.05) (Fig. 4C).

### FGFR1 mediates rTM effect on autophagy

**Figure 5:**
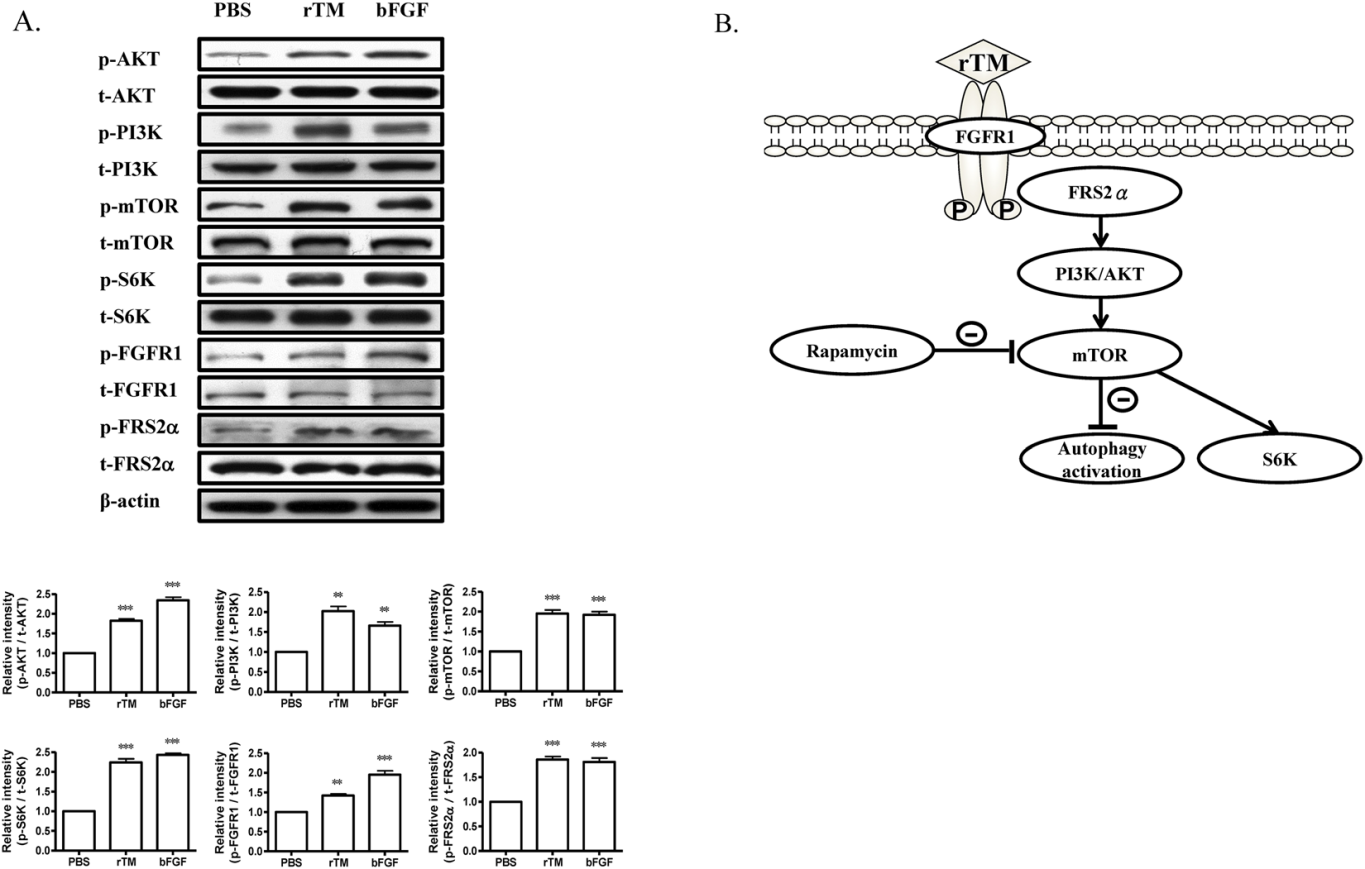
Signaling pathways of rTM effect on autophagy. (**A**) Western blot analyses were performed in ECs treated with PBS, rTM 100 ng/mL or bFGF 100 ng/mL for 24 hr. Bar graphs showed the ratios of protein expression levels at 24 hr in ECs treated with rTM or bFGF to PBS. Data are shown as mean ± SEM (n = 3). **p < 0.01 and ***p < 0.001 vs. cells treated with PBS. (**B**) Scheme of the signaling pathway for rTM to attenuate autophagy. By interacting and activating with FGFR1 and its downstream adapter protein, FRS2α in ECs, rTM activates AKT/mTOR pathways to suppress autophagy.

Finally, we looked at the upstream signaling pathway of rTM effect on autophagy. Our previous study showed that rTM directly interacted with FGFR1 and induced the activation of FGFR1^14^. Figure 5A shows that rTM activated FGFR1 and FGFR1 substrate, FGFR substrate 2α (FRS2α), a major adapter protein bound to FGFR juxtamembrane domains, with subsequent AKT/mTOR pathway activation and suppressed autophagy activity (Fig. 5A).

## Discussion

The major findings of this study were: (1) rTM suppressed stress-induced autophagy overactivation in ECs; (2) autophagy modulated ECs cell behavior; (3) autophagy was activated in aortic ECs in human atherosclerosis and apoE deficient mice; (4) rTM treatment suppressed aortic ECs autophagy and decreased atherosclerosis in apoE deficient mice and (5) rTM effect on autophagy was achieved through mTOR activation via the FGFR1/FRS2α-mediated PI3K/AKT pathway.

Endothelial dysfunction is one of the most critical initiating and perpetuating events during atherosclerotic plaque formation. Any treatment that could protect vascular endothelium may lead to atherosclerosis alleviation. We and other studies showed that rTM treatment could inhibit atherosclerosis in various animal models^17–19^. Initially, we considered the rTM’s thrombin inhibitory effects with thrombin-activated PAR-1 signal downregulation and reduced endothelial inflammation were the major mechanisms^19^. Recently, rTM was found to have direct endothelial protective effects. Treatment with rTM not only increases EC proliferation, migration and tubule formation but also reduces stress-induced EC apoptosis^11–13, 20^. In the current study, we further demonstrated that rTM suppressed stress-induced autophagy overactivation which could modulate ECs cell behaviors, and this function was one of the mechanisms that explain the rTM’s anti-atherosclerosis effect. Autophagy could either promote or suppress ECs migration or survival in different environmental conditions. Transient exposure (4 hr) to nutrient deprivation induced ECs autophagy and inhibition of this autophagy activation significantly reduced ECs tubule formation, migration and angiogenesis^21^. Similarly, stress with short-term hypoxia (30 min to 2 hr) induced autophagy in endothelial progenitor cells (EPCs) and inhibition of autophagy increased cell apoptosis^22^. These data indicated that transient stress-induced autophagy plays a protective role for cell proliferation and survival. However, autophagy could have the opposite effects for ECs if the stress is overwhelming or persistent. Chen *et al*. found that transient hypoxia-induced autophagy promoted ECs survival and growth, and this autophagy was regulated by hypoxia-inducible factor-1α (HIF-1α); but if the stress was prolonged (24 to 72 hr), autophagy overactivation became mTOR-dependent and caused EC death^7^. Brief hypoxia (2 hr)-induced autophagy could increase EPCs migration and tubule formation^23^. But under normal condition, EPCs or other cells without autophagy with ATG5 knockdown showed an increased migratory ability^24, 25^ and this finding was also observed in our studies. All the data showed that the environmental conditions play an important role in the cell response to autophagy.

In human and mouse atherosclerosis, autophagy activation in aortic ECs was found in our study. In vascular ECs, sustained autophagy triggers, such as oxidized low-density lipoprotein, advanced glycation end product, or reactive oxygen species, could come from circulating blood or within the subendothelial layer of atherosclerotic plaque^26^. The persistent autophagy overactivation leads to ECs apoptosis and endothelial dysfunction which may accelerate atherosclerotic process. Our *in vivo* experimental results demonstrated that rTM treatment could suppress ECs autophagy, decrease ECs apoptosis and atherosclerotic plaque area in the apoE deficient mice. Treatment with rTM could protect ECs by modulating mTOR-dependent autophagy and restrict atherosclerosis development. Other compound that activated mTOR and suppressed autophagy also showed anti-atherosclerotic effect in apoE deficient mice^27^. These data implied that manipulation of autophagy during atherosclerosis formation may carry therapeutic potential. However, the results showed in our animal experiments were the summation effect of rTM. The rTM effects on autophagy in other vascular cell types, such as smooth muscle cell and macrophage, need further studies. Activated mTOR is the major regulator to downregulate autophagy. We proved that rTM could inhibit autophagy via the activation of AKT/mTOR pathway and decrease expression of autophagy-related proteins and autophagosome formation in ECs. Our recent study showed that rTM protective effect on ECs was through FGFR1 activation. There was direct interaction between rTM and FGFR1 and the rTM effect can be inhibited by an FGFR1-specific tyrosine kinase inhibitor, PD173074, or by knockdown of FGFR1 using siRNA^14^. FRS2α is a membrane-anchored adaptor protein that is required in the FGF/FGFR1 signal transduction pathway. Previous studies showed that FRS2α is necessary for FGF to activate mTOR and suppress the autophagic activity in fibroblasts and cardiac progenitor cells^28, 29^. In the current study, we reconfirmed that rTM works through the similar transduction pathway by activating FGFR1/ FRS2α and AKT/mTOR to inhibit autophagy in ECs. A schematic overview of the transduction pathway of rTM and autophagy was proposed in the Fig. 5B.

In conclusion, we found that rTM activates AKT/mTOR by interacting with FGFR1/ FRS2α in vascular ECs. Activation of mTOR inhibits stress-induced EC autophagy overactivation and suppresses EC apoptosis. The EC protective effects contribute to the rTM’s anti-atherosclerosis function.

## Methods

### Recombinant TM protein

We used recombinant TM protein containing domain 2 and 3 (rTM) to perform the following experiments. The methods of expression and purification of rTM in the *Pichia pastoris* expression system used in our laboratory was previously described^13^.

### Human umbilical vein ECs culture

HUVECs (Invitrogen) were used between passages 2 and 4. HUVECs were cultured in M199 medium (Gibco, Grand Island, NY) supplemented with 20% FBS (Gibco), 1% endothelial cell growth supplement (Sigma-Aldrich, St. Louis, MO), 1% heparin and 1% penicillin/streptomycin (Sigma-Aldrich) at 37 °C.

### Western blot analyses

Western blots were performed with antibodies against AKT, p-AKT (Ser473), mTOR, p-mTOR (Ser2448), S6K, p-S6K (Ser235/236), PI3K, p-PI3K (Ser249), ATG5, LC3, FGFR1, p-FGFR1(Tyr653/654), FRS2α and p-FRS2α (Tyr196) (all from Cell Signaling, Danvers, MA). Total protein (30 μg) from each sample was loaded into a SDS-polyacrylamide gel. The separated proteins were transferred to a PVDF membrane, and the membrane was blocked with 5% skim milk, followed by incubation with a 1:1000 dilution of the indicated antibody. Antibody binding was detected with HRP-conjugated secondary antibodies. Bands were visualized with ECL and Kodak GBX developer/fixer. The blots were exposed to X-ray film (Fujifilm Medical, Stamford, CT). For quantitation, densitometric analysis of the immunoblot was performed using AlphaImager 2200 digital imaging system (Digital Imaging System, San Leandro, CA).

### Autophagosome immunofluorescence staining

Monodansylcadaverine (MDC) is an autofluorescent compound that can label autophagosome directly. Cells were stained with 50 μM MDC (Sigma-Aldrich) for 30 min at 37 °C and the fluorescent signals were captured using a confocal microscope (FV1000, Olympus, Japan). LC3 is located on the autophagosomal membrane and can be regarded as a specific autophagosome marker. To assess LC3 localization, the cells were incubated with LC3 antibody (1:500, MBL, Nagoya, Japan) and then incubated with Alexa Fluor 488 goat anti-rabbit IgG (1:200, Invitrogen). Cells were counterstained with 4′,6-diamidino-2-phenylindole (DAPI, Sigma-Aldrich) to identify the nuclei. Images were captured with the same confocal microscope. For quantitating autophagosomes, ten cells were randomly selected on the microscope (600X) and the number of autophagosome (MDC-positive dot) or LC3 puncta in each cell was counted in a magnified view (2400X) on the microscope. Data were presented as the average number of autophagosome or LC3 puncta per cell.

### ATG5 knockdown

Knockdown of ATG5 in HUVECs was performed with DharmaFECT siRNA transfection kit (Thermo Scientific, Lafayette, CO). ATG5 small interference RNA (siRNA) was a pool of 3 target-specific 19 nucleotide siRNAs designed to knock down the gene expression of human ATG5 (sc-41445; Santa Cruz Biotechnology, Santa Cruz, CA). Control siRNA (sc-37007, Santa Cruz Biotechnology) was used as a negative control. Cells treated with ATG5 siRNA or control siRNA were incubated for 72 hr before following experiments. The knockdown efficiency of ATG5 was analyzed by western blot. We used control or ATG5 siRNA at a concentration of 50 nM for all following experiments.

### ECs apoptosis assay

Based on the time- and rTM concentration-dependent stress results (Supplement Figure I), we used 24 hr as the SS time and 100 ng/mL as the rTM treatment concentration. Apoptosis was determined in the following groups of cultured ECs: (1) cells received normal media and without SS; (2) cells received SS and treated with PBS for 24 hr; (3) cells received SS and treated with rTM 100 ng/mL for 24 hr and (4) cells received SS and treated with rTM 100 ng/mL and rapamycin 10 µg/mL for 24 hrs. The cells were collected and apoptosis was evaluated by quantification of DNA fragmentation using the cell death detection ELISA kit (Roche Diagnostics, Mannheim, Germany). We also used flow cytometry to determine apoptosis. Cells were double stained with annexin V-fluorescein isothiocyanate (FITC) and propidium iodide (PI) by using FITC annexin V apoptosis detection kit (BD Biosciences, Franklin Lakes, NJ). The cells were analyzed by a fluorescence-activated cell sorter (FACSort, BD Biosciences).

### ECs migration and proliferation assay

For wound healing assay, cells treated with ATG5 siRNA or control siRNA were seeded and grew until nearly 100% confluence. A linear scratch was made by using 200 μL pipette tip. Then the cells were incubated for 24 hr and images were taken at 0 and 24 hr. For proliferation assay, cells were seeded in the 96 well plate (5000 cells per well) and transfected with ATG5 siRNA for 72 hr. After transfection, 10 μM BrdU was added to the cells and followed by incubation for 2 hr. The nuclear incorporation of BrdU was measured with a cell proliferation ELISA kit (Roche Diagnostics).

### Immunofluorescent staining of human aortas

Human aorta specimens were obtained from a recipient of cardiac transplantation with ischemic cardiomyopathy and severe coronary artery disease. Normal human aorta specimens obtained from a donor of cardiac transplantation with normal coronary artery were used for comparison. Human tissue collection was approved by our Institutional Review Board (IRB approval number: A-ER-103-043). For double immunofluorescent staining, the sections were stained for anti-human CD31 (1:50; GeneTex, Irvine, CA) and anti-LC3 (1:500; MBL) or anti-ATG13 (1:200; Sigma-Aldrich) antibodies, followed by incubation with respective fluorescent secondary antibodies (1:200; Invitrogen). Isotype IgG control antibodies were used as negative controls to confirm the specificity of respective primary antibodies (Calbiochem, San Diego, CA) in human and mouse aortic specimens (Supplement Figure II). Nuclei were counterstained with DAPI. The fluorescent signals were captured using a fluorescent microscope (BX61, Olympus).

### ApoE deficient mice

ApoE deficient (Jackson Laboratory, stock number: 002052; ApoE^−/−^ knockout C57BL/6 background, 8-week-old) mice were purchased from the Jackson Laboratory (Bar Harbor, ME) and fed with a high cholesterol diet (PMI LabDiet 40097, Richmond, IN) from 8 to 28 weeks of age. Initially, osmotic pumps (Alzet 2004; Durect, Cupertino, CA) containing rTM (145 μg/kg/day) were implanted subcutaneously on the back of mice for 4 weeks (from 8 to 12-week-old). At 28 week, the mice were sacrificed, the periaortic tissue was trimmed around the aorta and the whole aorta was obtained. The atherosclerotic plaques in aortas were stained with Oil-red O (Sigma-Aldrich). The images of the aortas were taken and analyzed by use of ImagePro Plus. The atherosclerotic severity was expressed as a percentage of atherosclerotic plaque area to the total aortic surface area. All animal experiments were performed in accordance with the institutional guidelines and approved by the Institutional Animal Care and Use Committee, National Cheng Kung University (Approval number:104055).

### Immunofluorescent staining of mouse tissue

The aortas isolated from mice were embedded in optimum cutting temperature compound. Frozen sections (5 μm thick) were fixed and blocked with 5% goat serum. For double-immunofluorescent staining, the sections were stained for anti-mouse CD31 (1:100; BD Biosciences) and anti-LC3 (1:500; MBL), anti-ATG13 (1:200; Sigma-Aldrich) or anti-pS6K (1:100; Cell Signaling) antibodies, followed by incubation with fluorescent secondary antibodies (1:200; Invitrogen). Nuclei were counterstained with DAPI. Terminal deoxynucleotidyl transferase-mediated dUTP nick-end labeling (TUNEL) assay was used to evaluate cell apoptosis with the *In Situ* Cell Death Detection kit (Roche Diagnostics). Images were captured with a fluorescence microscope (BX61, Olympus) and images were analyzed using Image J software. The intensities of immunofluorescence staining images were analyzed by AlphaImager 2200 software.

### Statistical Analysis

Data were presented as mean ± standard error. Comparisons between 2 groups were made by Mann Whitney test. For multiple comparisons of groups, one-way ANOVA was used and followed by Bonferroni post hoc analysis. All statistical analyses were performed using SPSS 12.0 (SPSS Inc. Chicago, Il, USA). A p value < 0.05 was considered statistically significant.

## Electronic supplementary material

Supplementary data

## References

[1] Deanfield JE, Halcox JP, Rabelink TJ (2007). Endothelial function and dysfunction: testing and clinical relevance. Circulation.

[2] Mizushima N, Levine B, Cuervo AM, Klionsky DJ (2008). Autophagy fights disease through cellular self-digestion. Nature.

[3] Choi AM, Ryter SW, Levine B (2013). Autophagy in human health and disease. The New England journal of medicine.

[4] Levine B, Kroemer G (2008). Autophagy in the pathogenesis of disease. Cell.

[5] Ravikumar B (2010). Regulation of mammalian autophagy in physiology and pathophysiology. Physiological reviews.

[6] Kim J, Kundu M, Viollet B, Guan KL (2011). AMPK and mTOR regulate autophagy through direct phosphorylation of Ulk1. Nature cell biology.

[7] Chen G (2013). Hypoxia-induced autophagy in endothelial cells: a double-edged sword in the progression of infantile haemangioma?. Cardiovascular research.

[8] Schrijvers DM, De Meyer GR, Martinet W (2011). Autophagy in atherosclerosis: a potential drug target for plaque stabilization. Arteriosclerosis, thrombosis, and vascular biology.

[9] Esmon CT (1989). The roles of protein C and thrombomodulin in the regulation of blood coagulation. The Journal of biological chemistry.

[10] Li YH, Kuo CH, Shi GY, Wu HL (2012). The role of thrombomodulin lectin-like domain in inflammation. Journal of biomedical science.

[11] Ikezoe T (2012). Thrombomodulin protects endothelial cells from a calcineurin inhibitor-induced cytotoxicity by upregulation of extracellular signal-regulated kinase/myeloid leukemia cell-1 signaling. Arteriosclerosis, thrombosis, and vascular biology.

[12] Chao TH (2014). Soluble thrombomodulin is a paracrine anti-apoptotic factor for vascular endothelial protection. International journal of cardiology.

[13] Shi CS (2005). Evidence of human thrombomodulin domain as a novel angiogenic factor. Circulation.

[14] Kuo CH (2015). FGFR1 mediates recombinant thrombomodulin domain-induced angiogenesis. Cardiovascular research.

[15] Torisu T (2013). Autophagy regulates endothelial cell processing, maturation and secretion of von Willebrand factor. Nature medicine.

[16] Breslow JL (1996). Mouse models of atherosclerosis. Science.

[17] Li JM (2004). Recombinant human thrombomodulin inhibits arterial neointimal hyperplasia after balloon injury. Journal of vascular surgery.

[18] Li YH (2006). Thrombomodulin plays an important role in arterial remodeling and neointima formation in mouse carotid ligation model. Thrombosis and haemostasis.

[19] Wei HJ (2011). Thrombomodulin domains attenuate atherosclerosis by inhibiting thrombin-induced endothelial cell activation. Cardiovascular research.

[20] Eguchi R (2014). Recombinant human soluble thrombomodulin attenuates FK506-induced endothelial dysfunction through prevention of Akt inactivation. Experimental cell research.

[21] Du J (2012). Role of autophagy in angiogenesis in aortic endothelial cells. American journal of physiology. Cell physiology.

[22] Wang HJ, Zhang D, Tan YZ, Li T (2013). Autophagy in endothelial progenitor cells is cytoprotective in hypoxic conditions. American journal of physiology. Cell physiology.

[23] Hu N (2015). Autophagy protein 5 enhances the function of rat EPCs and promotes EPCs homing and thrombus recanalization via activating AKT. Thrombosis research.

[24] Rhoads JM, Niu X, Odle J, Graves LM (2006). Role of mTOR signaling in intestinal cell migration. American journal of physiology. Gastrointestinal and liver physiology.

[25] Li WD (2015). Autophagy inhibits endothelial progenitor cells migration via the regulation of MMP2, MMP9 and uPA under normoxia condition. Biochemical and biophysical research communications.

[26] Martinet W, De Meyer GR (2009). Autophagy in atherosclerosis: a cell survival and death phenomenon with therapeutic potential. Circulation research.

[27] Peng N (2014). An activator of mTOR inhibits oxLDL-induced autophagy and apoptosis in vascular endothelial cells and restricts atherosclerosis in apolipoprotein E(−)/(−) mice. Scientific reports.

[28] Lin X (2011). FRS2alpha is essential for the fibroblast growth factor to regulate the mTOR pathway and autophagy in mouse embryonic fibroblasts. International journal of biological sciences.

[29] Zhang J (2012). FRS2alpha-mediated FGF signals suppress premature differentiation of cardiac stem cells through regulating autophagy activity. Circulation research.

