# Supplementary material for: Recombinant Thrombomodulin Exerts Anti-autophagic Action in Endothelial Cells and Provides Anti-atherosclerosis Effect in Apolipoprotein E Deficient Mice
Source: Sci Rep. 2017 Jun 12;7:3284. doi: 10.1038/s41598-017-03443-z (PMC5468323; doi:10.1038/s41598-017-03443-z)
Supplement: Supplementary file 1 — Supplementary data [file 41598_2017_3443_MOESM1_ESM.doc]

**Recombinant** [**Thrombomodulin Exerts Anti-autophagic Action in Endothelial Cells and Provides**](http://www.ncbi.nlm.nih.gov/pubmed/26302768) **Anti-atherosclerosis Effect in Apolipoprotein E Deficient Mice**

Po-Sheng Chen; Kuan-Chieh Wang; Ting-Hsing Chao; Hsing-Chun Chun^1^; Shi-Ya Tseng; Chawn-Yau Luo; Guey-Yueh Shi; Hua-Lin Wu; Yi-Heng Li

**Supplement Figure I**

A.

**Starvation (hr) 0 2 4 6 16 24**

**p-AKT**

**t-AKT**

**p-mTOR**

**t-mTOR**

**p- S6K**

**t- S6K**

**ATG5**

**LC3 I/II**

**β-actin**


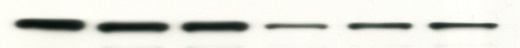

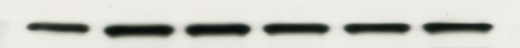

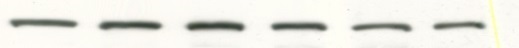

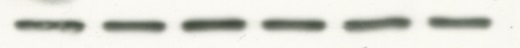

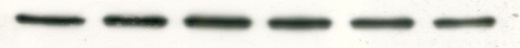

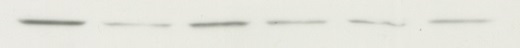

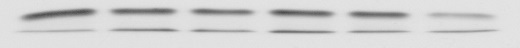

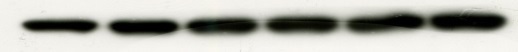

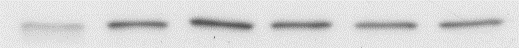


B.

**rTM (ng/mL) 0 10 50 100 250**

**p-AKT**

**t-AKT**

**p-mTOR**

**t-mTOR**

**p- S6K**

**t- S6K**

**ATG5**

**LC3 I/II**

**β-actin**


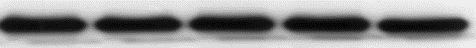

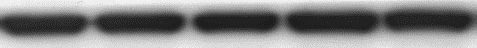

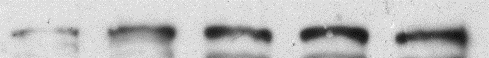

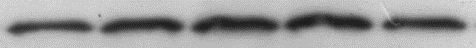

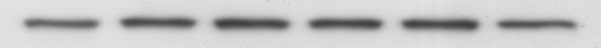

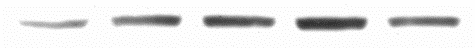

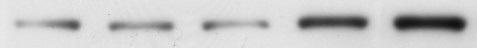


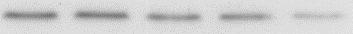


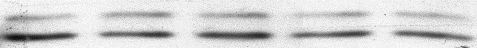


**Supplement Figure I. The time- and rTM concentration-dependent stress results.**

(A) Cultured ECs were stressed with SS from 0 to 24 hr. (B) Cultured ECs were treated with rTM from 0 to 250 ng/mL and stressed with SS for 24 hr. After indicated treatment, western blot analyses were performed for signaling pathway proteins AKT, p-AKT (Ser473), mTOR, p-mTOR (Ser2448), S6K, p-S6K (Thr389) and autophagy-related proteins, ATG5 and LC3.

**Supplemental Figure II**

A.


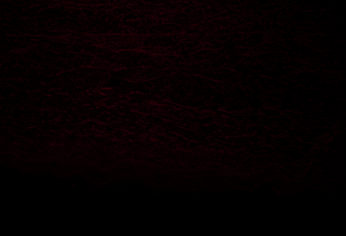

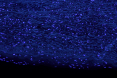

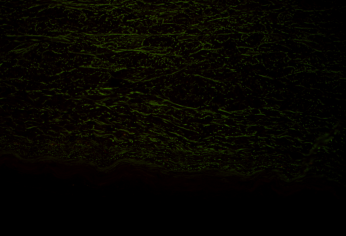

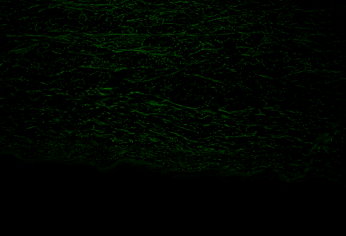


L

L

L

Rabbit IgG

Mouse IgG

Merge

B.


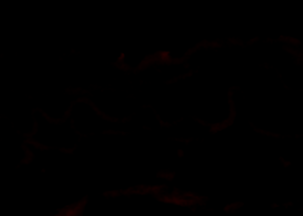

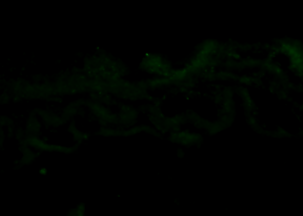

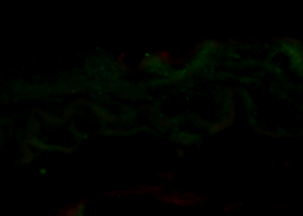


L

L

L


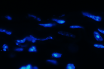


Rabbit IgG

Mouse IgG

Merge

**Supplement Figure II. Negative controls of immunostaining in human and mouse histological specimens**. Representative microscopic images of immunostaining with respective isotype-matched IgG controls in (A) human aortic specimen (in comparison with Figure 3) and (B) mouse aortic specimen (in comparison with Figure 4A). Nuclei are counterstained with DAPI. L, lumen. Scale bar, 200 μm in (A) and 50 μm in (B).
